# Review of Kissing Bugs (Hemiptera: Reduviidae: Triatominae) from China with Descriptions of Two New Species †

**DOI:** 10.3390/insects14050450

**Published:** 2023-05-10

**Authors:** Yisheng Zhao, Mingyuan Fan, Hu Li, Wanzhi Cai

**Affiliations:** Department of Entomology and MOA Key Lab of Pest Monitoring and Green Management, College of Plant Protection, China Agricultural University, Beijing 100193, China; zhaoyisheng@cau.edu.cn (Y.Z.); bs20203190917@cau.edu.cn (M.F.); tigerleecau@hotmail.com (H.L.)

**Keywords:** Chinese Triatominae, taxonomy, genitalia, key

## Abstract

**Simple Summary:**

Triatominae, commonly known as kissing bugs, are blood-feeding insects that can carry the parasite *Trypanosoma cruzi* to spread Chagas disease. In this study, we conducted a taxonomic review of Chinese triatomines, which involved describing two new species, *Triatoma picta* Zhao & Cai sp. nov. and *T. atrata* Zhao & Cai sp. nov. We also calculated the pairwise genetic distances of *Triatoma* and offered a key to species of Triatominae in China. This review may facilitate a better understanding of the diversity of Chinese kissing bugs.

**Abstract:**

Triatominae, the only blood-sucking subfamily in Reduviidae, are the vectors of Chagas disease. The majority of them are distributed in the Americas, while the diversity in China has been underestimated, as only two species have been recorded. Here, we describe two new species from China, *Triatoma picta* Zhao & Cai sp. nov. and *T*. *atrata* Zhao & Cai sp. nov., and provide a redescription of *T. sinica* Hsiao, 1965, along with remarks on *T. rubrofasciata* (De Geer, 1773). To facilitate the identification, we include photos, especially of genitalia, as well as a distribution map and a key to Chinese triatomines. We calculated the pairwise genetic distances between 23 *Triatoma* species, which further supported the validity of these new species. We anticipate that our taxonomic review will be useful for identifying Chinese Triatominae.

## 1. Introduction

Triatominae, commonly known as kissing bugs, are the only hematophagous subfamily of Reduviidae [1]. They are capable of transmitting Chagas disease by carrying the parasite *Trypanosoma cruzi*. Chagas disease is a potentially life-threatening illness, which infects an estimated six to seven million people worldwide [2] and causes about 12,000 deaths annually [3]. Efforts to control the spread of the disease as a public health problem have been ongoing for many years, and taxonomic research on vectors may be one such effort. 

Within the Triatominae subfamily, there are 154 extant species classified into eighteen genera and five tribes [4,5,6,7]. The largest and most diverse tribe is Triatomini Jeannel, 1919, which contains over half of the subfamily’s species and at least six extant genera [4,8]. Among these genera, *Triatoma* Laporte, 1832 is the biggest and the type genus. It is also the only genus that is widely dispersed, occurring on all continents except Europe and Antarctica. In Asia, *Triatoma* included seven species, two of which are found in China. *Triatoma rubrofasciata* (De Geer, 1773) has a global distribution, while *Triatoma sinica* Hsiao, 1965 is an endemic species which was only collected in Nanjing, China [9]. It was first described by Hsiao, but his original description was written in Chinese and lacked a detailed description of the genitalia [10]. Few taxonomic studies about this subfamily have been conducted in China. Qian et al. reported the presence of four *Triatoma* species in China [11]. However, two of them, *Triatoma pugasi* Lent, 1953 and *Triatoma migrans* Breddin, 1903, are not from China but rather from Java and Sumatra, as cited in their references, which clearly indicates an error in their report [11]. Therefore, a review of Triatominae in China is necessary. 

Currently, certain molecular biology techniques provide assistance in resolving taxonomic issues, including species identification. DNA barcoding is considered a method that can separate close relatives at the species level by using a short section of DNA from a specific gene or genes [12]. The most widely used barcode region for insects is a portion of the cytochrome c oxidase subunit I (COI) gene, ~658 base pairs (bp), found in mitochondrial DNA [12,13]. To calculate the genetic distances of this region between species, the Kimura two-parameter (K2P) model is frequently employed as a nucleotide substitution model [14,15,16]. Such methods have already been applied to distinguish Triatominae and may contribute significantly to our understanding of their biodiversity [17,18]. 

The objective of this study was to conduct a comprehensive review of Chinese triatomines, with the aim of identifying Chinese kissing bugs and gaining a better understanding of their diversity. Through our work, we identified two previously unknown species, *T*. *picta* Zhao & Cai sp. nov. and *T. atrata* Zhao & Cai sp. nov. We also redescribed *T. sinica* with two more specimens from Taiwan and offered some remarks about *T. rubrofasciata*. Illustrations of the habitus, body parts, and genitalia were provided. Additionally, we sequenced the barcode region of COI for four species (*T. picta* Zhao & Cai sp. nov., *T. atrata* Zhao & Cai sp. nov., *T. rubrofasciata*, and *T. migrans*) and calculated pairwise genetic distances among 23 *Triatoma* species to investigate the divergences between new species and other congeners. We also developed a key and a distribution map for Chinese triatomines. We expect our findings will contribute to a better understanding of the diversity and distribution of this important group of insects, Triatominae.

## 2. Materials and Methods

### 2.1. Specimens

All specimens were deposited in the Entomological Museum of China Agricultural University, Beijing, China (CAU). Specimens used for DNA barcoding were collected by light trapping between 2016 and 2020 (Table S1), and were preserved in 100% ethanol at—20 °C. The distribution of four species from China was mapped based on the collection sites of specimens.

### 2.2. Dissections and Measurements

The pygophores were removed from specimens and soaked in 100% lactic acid overnight, followed by boiling in ~20% lactic acid solution for ~40 min. Dissections were performed under a Motic binocular dissection microscope (Olympus SZX7), using a pair of forceps and an insect pin to separate the phallus from pygophores and stretch the endosoma. A detailed description of the dissection procedure can be found in our previous study [19]. All dissected genitalia were preserved in glycerol in plastic tubes which were pinned under the corresponding specimens. Measurements were taken using a calibrated micrometer and recorded in millimeters. 

### 2.3. Images and Image Processing

Habitus images of the specimens were captured using a Canon EOS 7D camera equipped with a 100 mm macro lens. For obtaining detailed images of heads, pronota, and scutella, a microscope (Nikon SMZ18) equipped with a Canon EOS 7D was used. Genital images were captured using an Olympus BX51 microscope coupled with a Canon EOS 7D camera. To produce images with an extended depth of field, a focus stacking technique was applied using Helicon Focus 5.3. The species distribution map was created using ArcMap 10.8. Finally, image plates were processed and edited using Adobe Photoshop 2020. 

### 2.4. Terminology 

The terminology adopted in this paper followed Lent and Wygodzinsky [8], Zhao et al. [19], Schuh and Weirauch [20], and Rosa et al. [21].

### 2.5. DNA Barcoding

DNA samples were extracted from tissues (thoracic muscles or leg muscles), which followed standard methods for DNA extraction using the dNeasy Blood and tissue kit (Qiagen). The COI genes were amplified with the primers LCO 1490 (5′-GGTCAACAAATCATAAAGATATTGG-3′) and HCO 2198 (5′-TAAACTTCAGGGTGACCAAAAAATCA-3′) [22]. Polymerase Chain Reaction (PCR) was conducted using Premix Taq™ (Ex Taq™ Version 2.0 plus dye), with the following cycling conditions: pre-denaturation at 94 °C for 3.5 min, denaturation at 94 °C for 30 s, annealing at 55 °C for 30 s, elongation at 72 °C for 1 min, and 35 cycles and elongation at 72 °C for 8 min. Sequences were bidirectionally sequenced using the same PCR primer pairs, and the sequence data have been deposited in GenBank. Our analysis included a total of 23 COI sequences from triatomines, of which 19 sequences were obtained from GenBank. Sequence alignment was carried out using Clustal W and edited using Geneious 10.1.3 (http://www.geneious.com/, accessed on 15 September 2019). Genetic distances were calculated using the Kimura two-parameter (K2P) model in MEGA X [23]. 

## 3. Results

### 3.1. Triatoma picta Zhao & Cai sp. nov. (Figure 1, Figure 2 and Figure 3)

#### 3.1.1. Diagnosis

This species closely resembles *T. migrans*, and they both have the following characters: a general body coloration of yellowish orange with dark markings; four dark brown stripes on the posterior lobe of the pronotum; a dark rectangular marking on each segment of the connexivum. However, it can be easily distinguished from *T. migrans* by its relatively straight lateral edges and the conical anterolateral angles of pronotum.

#### 3.1.2. Description 

**Figure 1:**
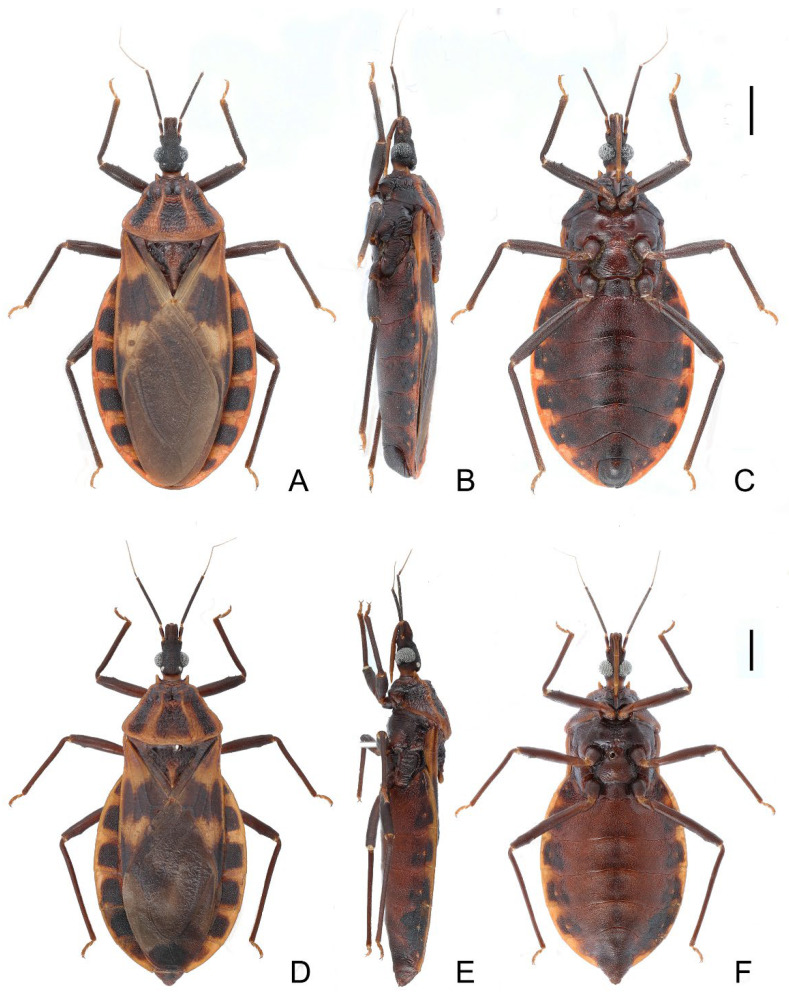
Habitus of *Triatoma picta* Zhao & Cai sp. nov. (**A**–**C**) holotype, male. (**D**–**F**) paratype, female. (**A**,**D**) dorsal side; (**B**,**E**) lateral side; (**C**,**F**) ventral side. Scale bars: 3.00 mm.

**Figure 2:**
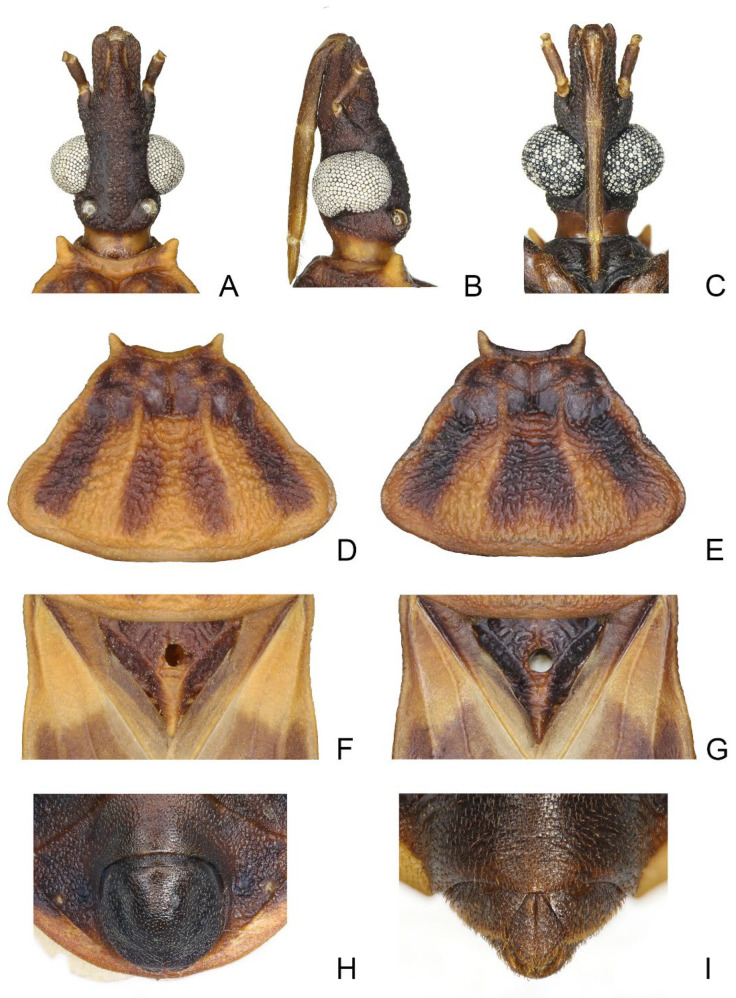
Head, pronotum, scutellum, genitalia of *Triatoma picta* Zhao & Cai sp. nov. (**A**–**C**) Head: (**A**) dorsal side; (**B**) lateral side; (**C**) ventral side. (**D**,**E**) two pronotum coloration patterns. (**F**,**G**) two scutellum coloration patterns. (**H**) male external genitalia. (**I**) female external genitalia.

Coloration. Body predominantly yellowish orange with dark markings (Figure 1). Head dark brown; neck yellowish orange (Figure 2A–C). Visible labial segments mostly yellowish orange; bottom of first visible labial segment dark brown (Figure 2B,C). First and second antennal segments dark brown, third and fourth segments yellowish (Figure 1). Inner edge of anterolateral angle of pronotum blurry dark brown; anterior lobe of pronotum generally brown with a pair of yellowish orange petal-like markings; posterior lobe with four longitudinal dark brown stripes, and two central stripes close together in some specimens (Figure 2D,E). Scutellum dark brown except central area and conical process yellowish orange (Figure 2F,G). Clavus mostly dark brown, apex of clavus yellowish in some specimens; apex of corium and central irregular spot dark brown; membrane brown (Figure 1A,D). Legs dark brown; tarsi yellowish. Each segment of connexivum with a dark brown rectangular spot. Sternites generally dark brown; spiracles with yellow narrow margins (Figure 1C–F). 

Structure. *Head*. Head elongated and granulose, ~1.5 × as long as width across eye (1:1.45–1.60), slightly shorter than length of pronotum (1:1.15–1.35) (Figure 1A,D); apex of maxillary plates slightly surpassing clypeus (Figure 2A–C); anteocular region ~3 × as long as postocular region in length (1: 2.65–2.88). Eyes relatively small, with width in dorsal view shorter than synthlipsis (1:1.45–1.94) (Figure 2A); in lateral view, eyes far away from upper surface of head and extending to lower surface (Figure 2B). Ratio of antennal segments 1:4.00–4.98:2.55–3.14:2.00–2.73. Third labial segment approaching anterior edge of pronotum; ratio of labial segments 1:1.19–1.28:0.39–0.44 (Figure 2B,C). *Thorax* (Figure 1A–F and Figure 2D–G). Anterolateral angles prominent, and sub-conical with sharp tips. Anterior region of pronotum granulose; posterior region wrinkled; length of posterior pronotal lobe ~2 × as long as anterior lobe (1:2.10–2.37), and ~1.5 × wider than anterior lobe (1:1.19–1.78); submedian carinae prominent and extending to half of posterior lobe; median longitudinal furrow of anterior lobe distinct; transverse furrow shallow; lateral edges relatively straight (Figure 2D,E). Scutellum triangular with a narrow Y-shaped ridge; conical process shorter than half of scutellum (Figure 2F,G). Pleura of meso- and metathoracices wrinkled (Figure 1B,E). Legs long and slender; apex of fore and mid tibiae with spongy fossae; two pairs of denticles at subapical of fore and mid femora in most specimens (Figure 1C,F). Hemelytra of male and female approaching tip of abdomen (Figure 1A,D).

**Figure 3:**
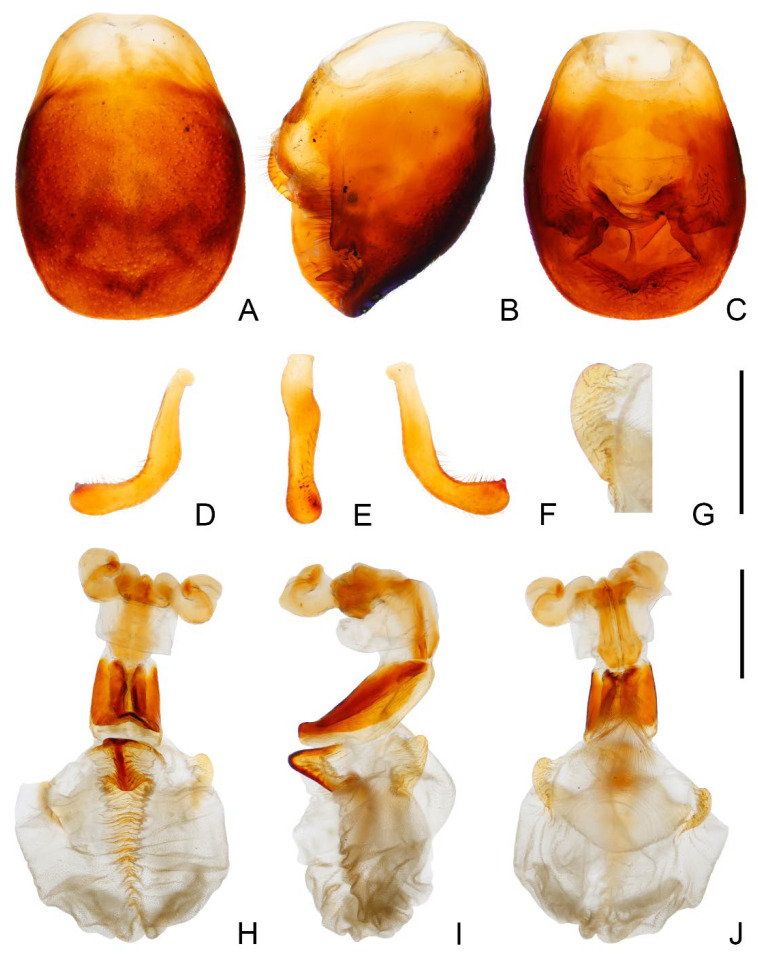
Male external genitalia of *Triatoma picta* Zhao & Cai sp. nov. (**A**–**C**) pygophore: (**A**) ventral view; (**B**) lateral view; (**C**) dorsal view. (**D**–**F**) paramere: (**D**) dorsal view; (**E**) lateral view; (**F**) ventral view. (**G**) basal lateral sclerite of endosoma. (**H**–**J**) everted phallus: (**H**) dorsal side; (**I**) lateral side; (**J**) ventral side. Scale bars: 1.00 mm (**A**–**F**,**H**–**J**) 0.50 mm (**G**).

*Genitalia*. Female external genitalia: posterior edge of VII sternite sinuous; VIII gonocoxites subtriangular, IX sternite visible (Figure 2I). Male external genitalia: transverse bridge of pygophore strongly sclerotized; median process of pygophore sharply pointed (Figure 3B,C). Paramere curved and with a denticle (Figure 3D–F). Width of arms of basal plate equal to that of transverse bridge of basal plate; basal plate extension approximately equal to arms of basal plate in length; dorsal phallothecal sclerite ellipsoid and tip raised in lateral view (Figure 3H,I). Medial basal sclerite of phallosoma strongly sclerotized and subtriangular in lateral view, and its upper surface flat (Figure 3H,I). Two lateral sclerites of endosoma ear-like without denticles (Figure 3G–J); surface of the endosoma granulose and not sclerotized; membrane in middle of dorsal surface of endosoma wrinkled and distinctly thicker than other membranous areas (Figure 3H). 

#### 3.1.3. Etymology

The species name is taken from Latin, picta, in allusion to its bright yellow color. 

#### 3.1.4. Distribution

**Figure 4:**
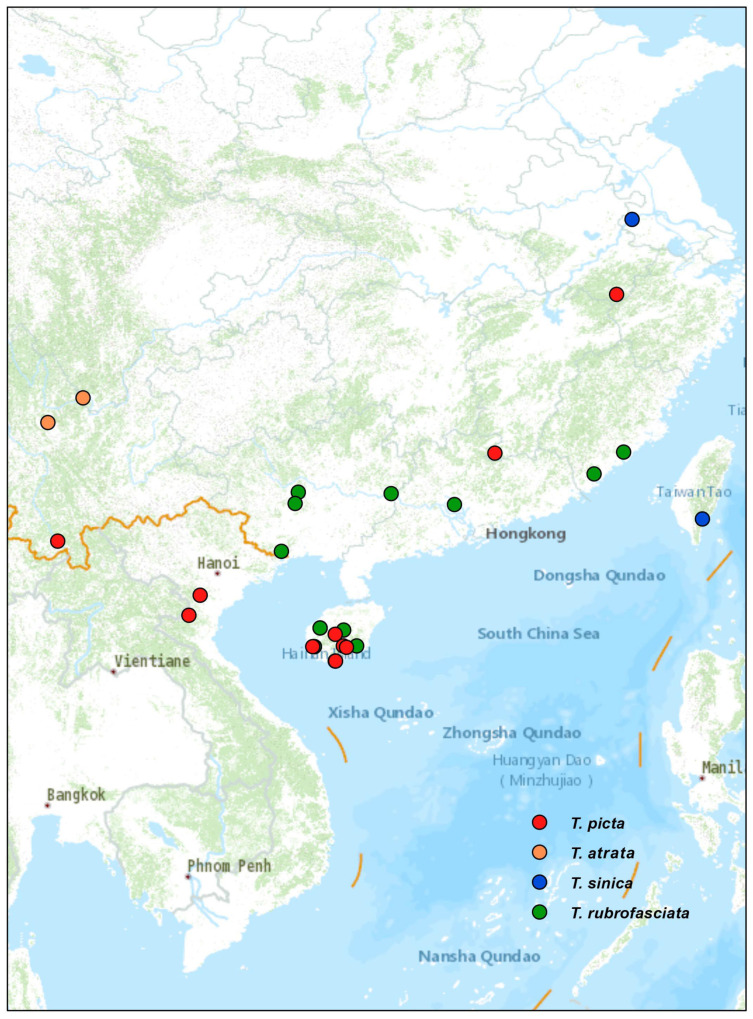
Distribution of four Triatominae species from China.

China (Anhui, Hainan, Jiangxi, Yunnan); Vietnam (Ninh Binh, Nghe An.) (Figure 4). 

#### 3.1.5. Measurements 

[in mm]: ♂ (n = 13) Total length to tip of abdomen 21.17–26.40. Length of head (excluding neck) 3.17–4.00; width of head 2.04–2.75; length of anteocular region 1.73–2.01; length of postocular region 0.60–0.75; width of eye 0.51–0.82; synthlipsis 0.99–1.19. Length of antennal segments I–IV = 0.64–0.85/3.19–3.70/2.01–2.35/1.75. Length of visible labial segments I–III = 1.44–1.70/1.84–2.15/0.64–0.72. Length of anterior lobe of pronotum 1.13–1.35; length of posterior pronotal lobe 2.58–3.20; width of anterior pronotal lobe 3.32–4.25; width of posterior pronotal lobe 5.35–7.55. Length of scutellum 2.32–3.25; width of scutellum 2.70–3.75. Length of hemelytron 14.14–17.69. Width of abdomen 7.85–10.75. 

♀ (n = 2) Total length to tip of abdomen 26.49–27.15. Length of head (excluding neck) 4.00–4.10; width of head 2.50–2.62; length of anteocular region 2.10–2.15; length of postocular region 0.75–0.81; width of eye 0.65–0.67; synthlipsis 1.20–1.29. Length of antennal segments I–IV = 0.85–1.00/3.95–4.00/2.55/1.92–2.00. Length of visible labial segments I–III = 1.80–1.85/2.15–2.20/0.70–0.75. Length of anterior lobe of pronotum 1.43–1.45; length of posterior pronotal lobe 3.00–3.40; width of anterior pronotal lobe 4.72–6.35; width of posterior pronotal lobe 6.80–7.55. Length of scutellum 2.90–3.15; width of scutellum 3.45–4.88. Length of hemelytron 18.15–18.50. Width of abdomen 10.75–11.14. 

#### 3.1.6. Materials Examined

Type material. Holotype: 1♂, China: Jianfeng Mountain, Ledong Li Autonomous County, Hainan, JY Wang [leg.], 23–25. IV. 2021. Paratype: 1♂, China: Jianfeng Mountain, Ledong Li Autonomous County, Hainan, ZQ Wang, YL Che, PM Wang [leg.], 25. VIII. 2002; 1♀, China: Qijia Village, Qiongzhong Li and Miao Autonomous County, Hainan, KY Zhang [leg.], 19.17° N, 109.71° E, 657 m alt., light trapping, 6. IV. 2021. 

Non-Type material. China: 1♀, Bawangling National Nature Reserve, Changjiang Li Autonomous County, Hainan, W Li [leg.], 11. VI. 2010; 4♂, Jianfeng Mountain, Ledong Li Autonomous County, Hainan, YS Zhao [leg.], 26. IV. 2016; 26. VIII. 2002; 24.II.1982; 10.VI.1983; 2♂, Bawangling National Nature Reserve, Changjiang Li Autonomous County, Hainan, MH Ke [leg.], 23–28. V. 1983; YC Zheng [leg.], IV.29. 2022; 1♂, Nakai Village, Yinggeling Natural Reserve, Baisha Li Autonomous County, Hainan, YS Zhao [leg.], 21.VI.2016; 1♂, Hongmao Village, Baisha Li Autonomous County, Hainan; YB Ba, JT Lang [leg.], 28–29.V.2007; 1♂, Linshui Li Autonomous County, Hainan, 28.III.1976; 1♂, Sanya, Hainan, 3. V. 1985; 1; 1♂, Jinuo Shan Zhai, Jinghong, Xishuangbanna Dai Autonomous Prefecture, Yunnan, ZT Wei [leg.]; 1♂, Quannan County, Jiangxi, ZX Huang [leg.], 8. VIII. 2019; 1♂, Tunxi, Anhui; VIII.1984. Vitnam: 2♂, Cuc Phuong, Gia Vien, Ninh Binh Prov. 370 m alt., light trapping, M. Tomokuni [leg.], 11. X. 1995; S. Uema [leg.], 27. 5. 1995; 1♂, Que Phong District, Nghe An, LT Ha [leg.], 7. VII. 1997. 

### 3.2. Triatoma atrata Zhao & Cai sp. nov. (Figure 5, Figure 6 and Figure 7)

#### 3.2.1. Diagnosis

*Triatoma atrata* Zhao & Cai sp. nov. can be distinguished from other *Triatoma* species by the following characters: black-colored body without obvious markings on the connexivum; a pronotum more than twice as wide as the head; nearly rounded rectangular lateral sclerites of endosoma with denticles. This species is most similar to *T. sinica* but can be differentiated by its small eyes and the absence of markings on the connexivum. 

#### 3.2.2. Description

**Figure 5:**
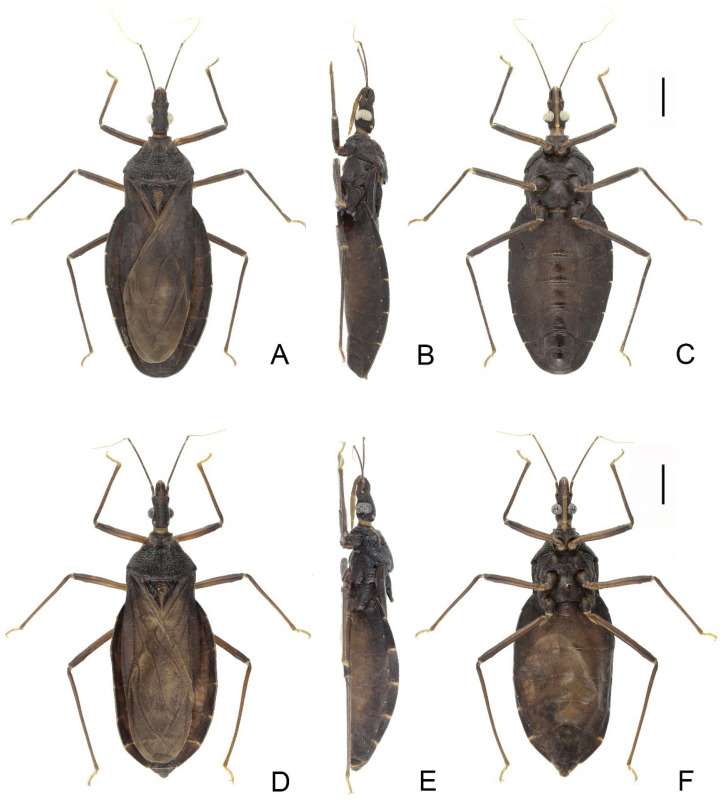
Habitus of *Triatoma atrata* Zhao & Cai sp. nov. (**A**–**C**) holotype, male. (**D**–**F**) paratype, female. (**A**,**D**) dorsal side. (**B**,**E**) lateral side. (**C**,**F**) ventral side. Scale bars: 3.00 mm.

**Figure 6:**
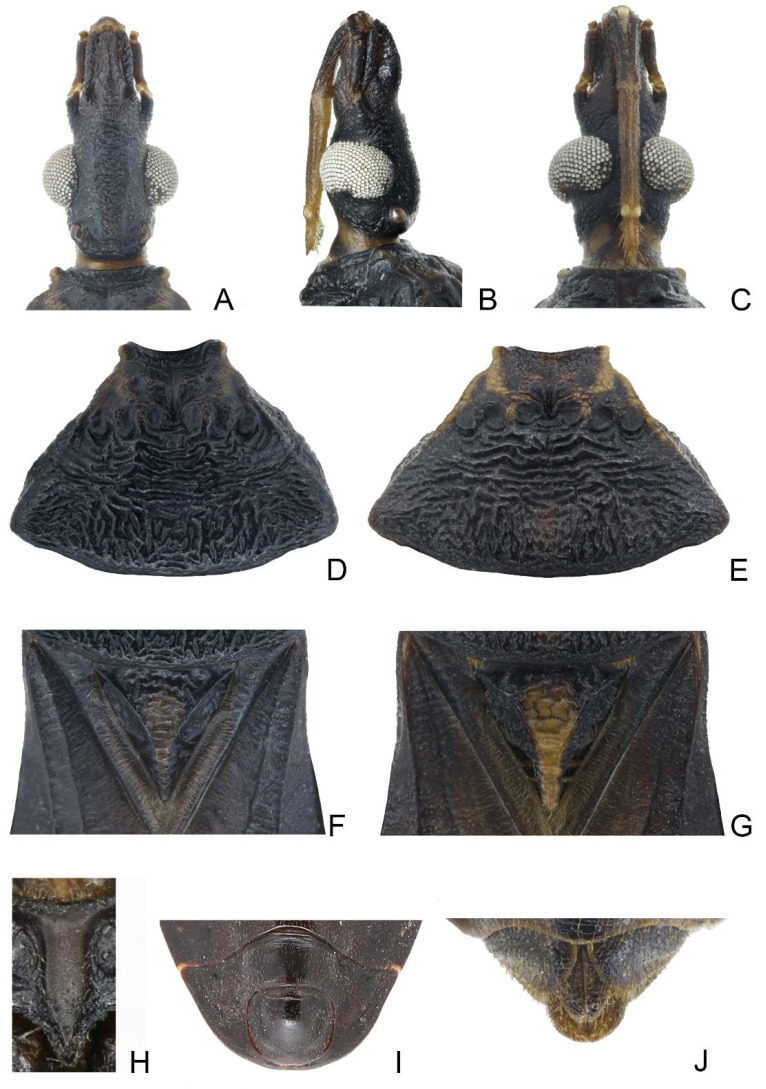
Head, thoracic structures, genitalia of *Triatoma atrata* Zhao & Cai sp. nov. (**A**–**C**) head: (**A**) dorsal side; (**B**) lateral side; (**C**) ventral side. (**D**,**E**) two pronotum coloration patterns. (**F**,**G**) two scutellum coloration patterns. (**H**) stridulatory sulcus. (**I**) male external genitalia. (**J**) female external genitalia.

Coloration. Body, mostly black with light yellow markings (Figure 5). Neck, second and third visible labial segments, third and fourth antennal segments, except bottom part, light yellow (Figure 5 and Figure 6A–C). Outer margins of anterolateral angles, lateral margin of anterior lobe of pronotum, and a pair of irregular markings on anterior lobe light yellow (Figure 6D,E). Central region and conical process of scutellum light yellow (Figure 6F,G). Forewing generally dark brown to black; apex of clavus, inner side of corium, and most of membrane light yellow (Figure 5A,D). Trochanters with blurry light-yellow spots (Figure 5C,F); femora dark brown to black; apex of tibiae light yellow. Joint area of adjacent segments of connexivum light yellow (Figure 5). Sternites generally dark brown to black; spiracles with light yellow narrow margins (Figure 5D–F). 

Structure. *Head*. Head elongated, ~1.5 × as long as width across eyes (1:1.76–1.81), slightly longer than length of pronotum (1:1.07–1.11). Apex of maxillary plate attaining level of apex of clypeus (Figure 6A). Anteocular region ~3 × as long as postocular region in length (1: 2.76–3.66) (Figure 6A). Eyes small, width of eye in dorsal view much smaller than synthlipsis (1:2.24–2.40); in lateral view, eyes far away from upper surface of head and close to lower surface (Figure 6A,B). Ratio of antennal segments 1:4.85–5.19:2.94–3.63:2.86–2.75. Third labial segment approaching to anterior edge of pronotum; ratio of labial segments 1:1.14–1.21:0.43–0.63 (Figure 6B,C). *Thorax*. Anterolateral angles, small with rounded tips. Surface of anterior lobe of pronotum granulose, and surface of posterior lobe wrinkled; length of posterior pronotal lobe ~2 × as that of anterior lobe (1:1.88–1.98); posterior pronotal lobe ~1.5 ×as wide as anterior lobe (1:1.60–1.66); submedian carinae not prominent; median longitudinal furrow of anterior lobe distinct; transverse furrow shallow; lateral edges relatively straight (Figure 6D,E). Scutellum triangular with narrow Y-shaped ridge; conical process shorter than half of whole scutellum (Figure 6F,G). Pleura of meso- and metathoracices wrinkled (Figure 5B,E). Stridulatory sulcus wide, length ~3 × as long as width (Figure 6H). Legs long and slender. Fore and mid femora mostly with two pairs of small subapical denticles and denticles on hind femur not distinct; apex of fore and mid tibiae with spongy fossae (Figure 5C,F). Hemelytra of male approaching to middle of eighth tergum, and that of female reaching to tip of the seventh tergum (Figure 5A,D). 

**Figure 7:**
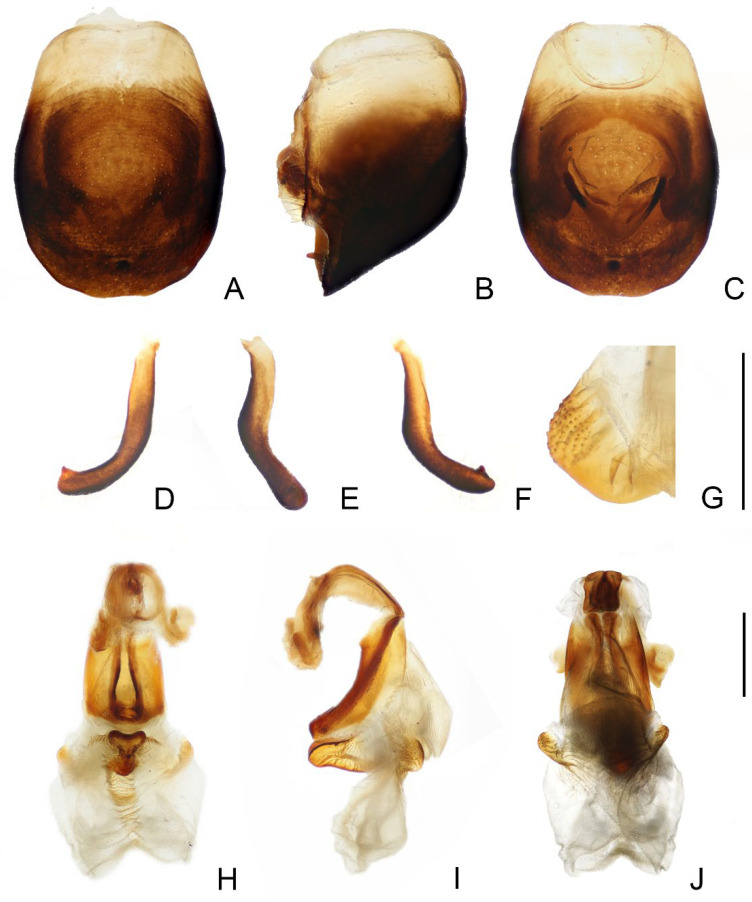
Male external genitalia of *Triatoma atrata* Zhao & Cai sp. nov. (**A**–**C**) pygophore: (**A**) ventral view; (**B**) lateral view; (**C**) dorsal view. (**D**–**F**) paramere: (**D**) dorsal view; (**E**) lateral view; (**F**) ventral view. (**G**) basal lateral sclerite of endosoma. (**H**–**J**) everted phallus: (**H**) dorsal side; (**I**) lateral side; (**J**) ventral side. Scale bars: 1.00 mm (**A**–**F**,**H**–**J**) 0.50 mm (**G**).

*Genitalia*. Female external genitalia: posterior edge of VII sternite sinuous; VIII gonocoxites subtriangular; IX sternite visible (Figure 6J). Male external genitalia: transverse bridge of pygophore strongly sclerotized; median process of pygophore long and slender (Figure 7B,C). Paramere curved with a denticle (Figure 7D–F). Width of basal plate arms equal to that of transverse bridge of basal plate; basal plate extension approximately equal to arms of basal plate in length (Figure 7H,I). Dorsal phallothecal sclerite ellipsoid with slightly raised tip. Medial basal sclerite of phallosoma strongly sclerotized and oblong in lateral view; its tip rounded and concaved in middle of upper surface (Figure 7H,I). Two lateral sclerites of endosoma rounded rectangular with denticles (Figure 7G); surface of endosoma with dense denticles or wrinkles and not sclerotized; membrane in middle of dorsal surface of endosoma wrinkled and distinctly thicker than other membranous areas (Figure 7H).

Measurements: [in mm] ♂ (n = 1) Total length to tip of abdomen 22.09. Length of head (excluding neck) 3.60; width of head 2.05; length of anteocular region 1.93; length of postocular region 0.70; width of eye 0.56; synthlipsis 0.93. Length of antennal segments I–IV = 0.80/3.88/2.35/2.00. Length of visible labial segments I–III = 1.60/1.93/0.70. Length of anterior lobe of pronotum 1.15; length of posterior pronotal lobe 2.20; width of anterior pronotal lobe 3.25; width of posterior pronotal lobe 5.40. Length of scutellum 2.50; width of sctellum 2.85. Length of hemelytron 15.37. Width of abdomen 8.30.

♀ (n = 2) Total length to tip of abdomen 23.20–23.30. Length of head (excluding neck) 3.55–3.70; width of head 1.96–2.05; length of anteocular 2.12–2.13; length of postocular 0.58–0.70; width of eye 0.45–0.48; synthlipsis 1.01–1.15. Length of antennal segments I–IV = 0.77–0.80/3.93–4.15/2.59–2.90/2.20. Length of visible labial segments I–III = 1.59–1.75/1.84–2.00/0.63–0.75. Length of anterior lobe of pronotum 1.16–1.20; length of posterior pronotal lobe 2.18–2.38; width of anterior pronotal lobe 3.20–3.50; width of posterior pronotal lobe 5.24–5.60. Length of scutellum 2.38–2.48; width of scutellum 2.97–3.00. Length of hemelytron 15.30–15.85. Width of abdomen 8.85–9.11.

#### 3.2.3. Etymology

The species name is taken from Latin, atrata, in allusion to its black color.

#### 3.2.4. Distribution

China (Sichuan, Yunnan) (Figure 4). 

#### 3.2.5. Materials Examined

Type material. Holotype: 1♂, China: Binchuan County, Dali Bai Autonomous Prefecture, Yunnan, 25.70° N, 100.55° E, light trapping, XJ Zhao [leg.], 8. VI. 2020. Paratype: 1♀, China: Binchuan County, Dali Bai Autonomous Prefecture, Yunnan, 25.70° N, 100.55° E, light trapping, XJ Zhao [leg.], 10. VIII. 2016; 1♀, China: Panzhihua, Sichuan, H Li [leg.], VII. 2014. 

### 3.3. Triatoma sinica Hsiao, 1965 (Figure 8, Figure 9 and Figure 10)

#### 3.3.1. Diagnosis

*Triatoma sinica* can be distinguished from its congeners by a combination of the following characters: yellowish lateral margins and a submedian carinae of the pronotum; small and conical anterolateral angles; each segment of the connexivum being brown or dark brown with wide transverse yellow stripes on the ~1/4 anterior region and ~1/4 posterior region; and median-sized eyes with a width equal to the synthlipsis. This species resembles *T. atrata* Zhao & Cai sp. nov. and *T. rubrofasciata*. It can be differentiated from *T. atrata* Zhao & Cai sp. nov. by having yellowing markings, as noted in the diagnosis of *T. atrata* Zhao & Cai sp. nov. *Triatoma sinica* can be distinguished from *T. rubrofasciata* by the presence of yellow markings on the connexivum and the short first antennal segment that does not reach the apex of the maxillary plate. 

#### 3.3.2. Redescripition

**Figure 8:**
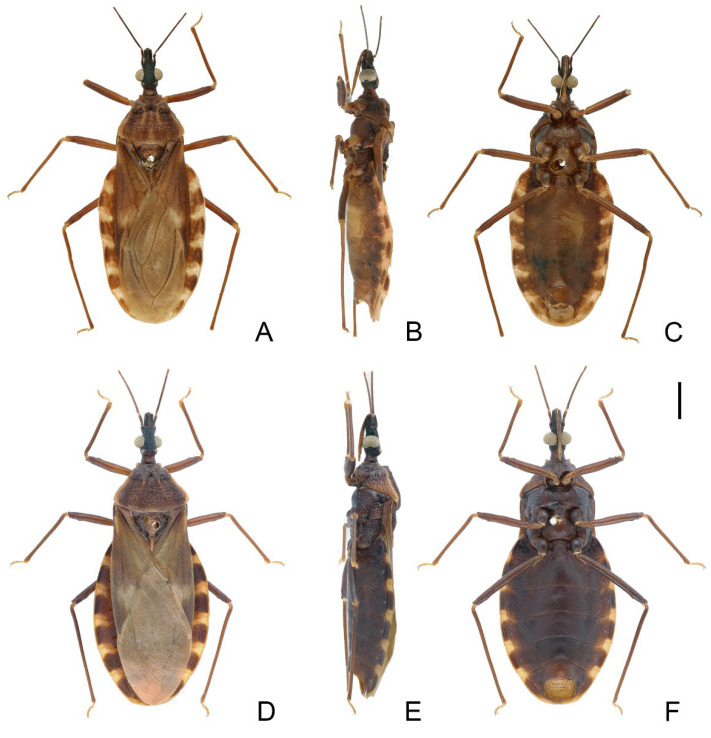
Habitus of *Triatoma sinica* Hisao, 1965, pygophore removed. (**A**–**C**) specimen collected in Nanjing. (**D**–**F**) specimen collected in Taiwan. (**A**,**D**) dorsal side; (**B**,**E**) lateral side; (**C**,**F**) ventral side. Scale bars: 3.00 mm.

**Figure 9:**
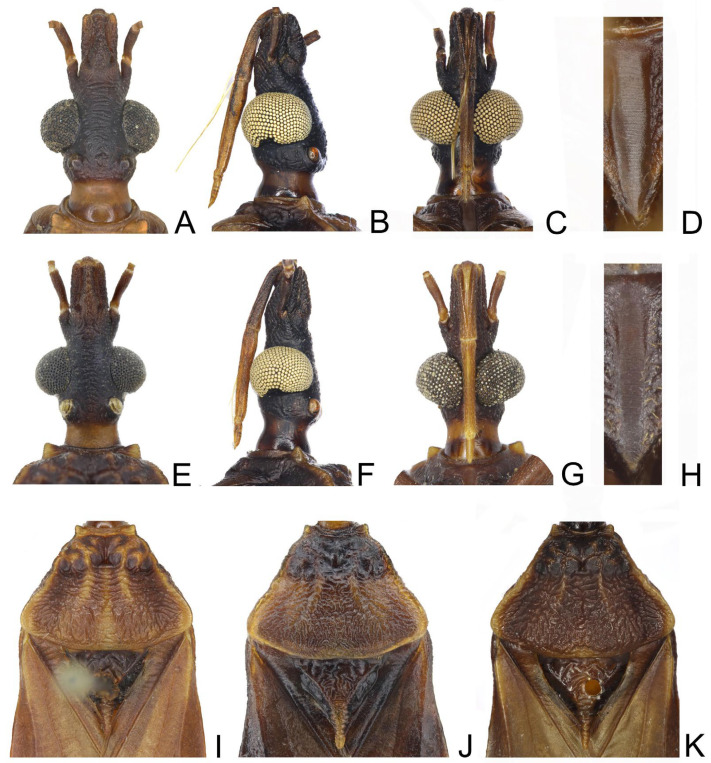
Head, thoracic structures, genitalia of *Triatoma sinica* Hisao, 1965. (**A**–**D**,**I**) specimen collected in Najing. (**E**–**H**,**J**,**K**) specimens collected in Taiwan. (**A**–**C**,**E**–**G**) head: (**A**,**E**) dorsal side; (**B**,**F**) lateral side; (**C**,**G**) ventral side. (**I**–**K**) three pronotum coloration patterns. (**D**,**H**) stridulatory sulcus.

Coloration. Body mostly brown to dark brown with yellowish stripes and markings (Figure 8). Neck light brown to yellow (Figure 9A–C). Visible labial segments yellowish except bottom area (Figure 9B,C,E–G). First and second antennal segments dark brown (Figure 8A–F). Anterolateral angles yellow; inner side of anterolateral angles of some specimens brown. Lateral margins of pronotum yellow; submedian carinae yellow or light brown; some specimens with a yellow longitudinal stripe on center of posterior lobe; anterior lobe of pronotum brown or dark brown with a pair of yellow or brown petal-like markings on anterior lobe; color of posterior lobe lighter than that of anterior lobe. Central region of scutellum brown, conical process of scutellum gradually yellow towards tip; outside of Y-shaped area dark brown or black (Figure 9I–K). Bottom of clavus brown; a stripe along R+M vein yellowish, and an irregular marking on the subapical corium light yellow, most membrane pale brown (Figure 8A,D). Legs brown to dark brown. Tarsi yellowish. Each segment of connexivum brown or dark brown with wide transverse yellow stripes on the ~1/4 anterior region and ~1/4 posterior region (Figure 8A–F). Sternites generally dark brown to black; spiracles with yellow narrow margins (Figure 8B,C,E,F). 

Structure. *Head*. Head elongated and central area of dorsal surface wrinkled; length of head ~1.5 × as long as width across eyes (1:1.53–1.58), and slightly shorter than length of pronotum (1:1.13–1.16) (Figure 8A,D and Figure 9A,E). Apex of maxillary plates extending to apex of clypeus (Figure 9A,E). Anteocular region ~3 × as long as postocular region in length (1: 2.75–2.85); eyes median size, width of eye in dorsal view equal to synthlipsis (1:1.01–1.07) (Figure 9A,E); in lateral view, eyes close to upper surface of head and passing over lower surface (Figure 9B,F). Ratio of antennal segments 1:4.45–4.83; third and fourth segments lost. Third labial segment approaching to anterior edge of pronotum; ratio of labial segments 1:1.32–1.45:0.36–0.48 (Figure 9B,C,F,G). *Thorax*. Anterolateral angles small and conical. Surface of pronotum wrinkled, and lateral edges of pronotum straight; length of posterior lobe ~2 × as that of anterior lobe (1:2.12–2.27); posterior pronotal lobe ~1.5 ×as wide as anterior lobe (1:1.56–1.60); submedian carinae extending to half of posterior lobe; median longitudinal furrow distinct; transverse furrow shallow (Figure 9I–K). Scutellum triangular with narrow Y-shaped ridge; conical process slender and shorter than half of whole scutellum (Figure 9I–K). Pleura of meso- and metathoracices wrinkled (Figure 8B,E). Stridulatory sulcus narrow, length of it ~5 × as long as width (Figure 9D,H). Legs long and slender. Fore and mid femora both with two pairs of proximal denticles in most specimens (Figure 8C,F). Hemelytra of male approaching tip of abdomen (Figure 8A,D). 

**Figure 10:**
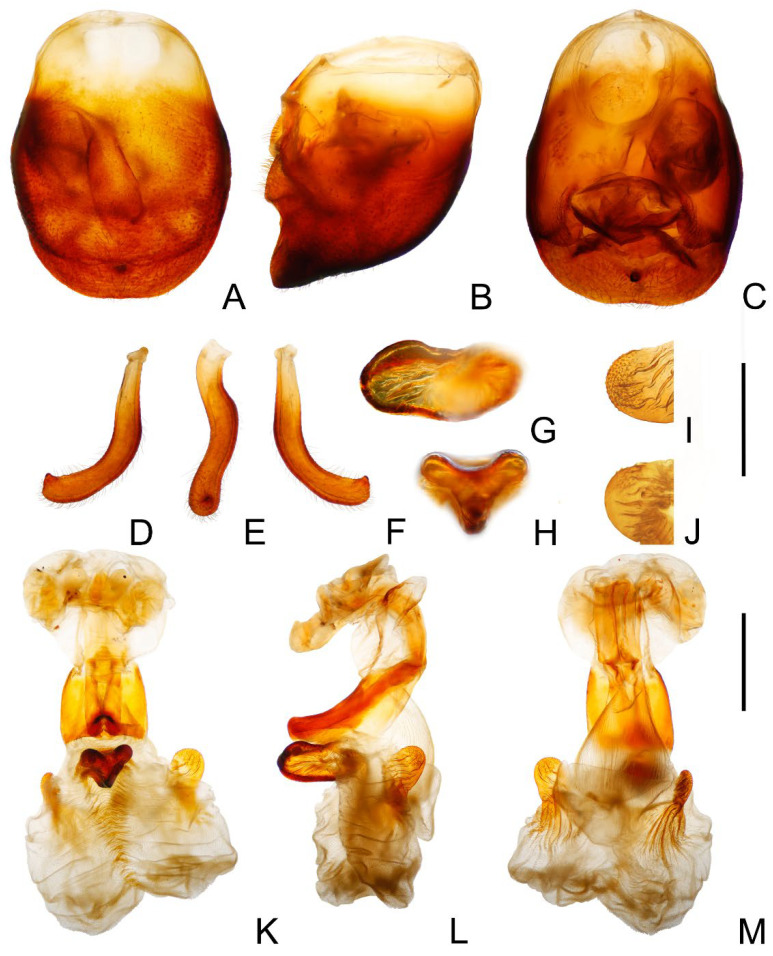
Male external genitalia of *Triatoma sinica* Hisao, 1965. (**A**–**F**,**I**,**K**–**M**) specimen collected in Taiwan. (**G**,**H**,**J**) specimen collected in Nanjing. (**A**–**C**) pygophore: (**A**) ventral view; (**B**) lateral view; (**C**) dorsal view. (**D**–**F**) paramere: (**D**) dorsalIew; (**E**) lateral view; (**F**) ventral view. (**G**,**H**) medial basal sclerite of phallosoma: (**G**) lateral side; (**H**) dorsal side; (**I**,**J**) basal lateral sclerite of endosoma. (**K**–**M**) everted phallus: (**K**) dorsal side; (**L**) lateral side; (**M**) ventral side. Scale bars: 1.00 mm (**A**–**F**,**K**–**M**) 0.50 mm (**G**–**J**).

*Genitalia*. Male external genitalia: Transverse bridge of pygophore strongly sclerotized and narrow; a pair of dorsal sclerites of genital opening large; median process of pygophore sharply pointed (Figure 10A–C). Paramere curved with a denticle (Figure 10D–F). Width of arms of basal plate equal to that of transverse bridge of basal plate (Figure 10K); basal plate extension roughly same length as arms of basal plate (Figure 10L); dorsal phallothecal sclerite oval with raised tip (Figure 10I); medial basal sclerite of phallosoma strongly sclerotized and oblong in lateral view, central of upper surface concaved (Figure 10G–H). Two lateral sclerites of the endosoma sub-semicircle with denticles (Figure 10I–J); surface of endosoma granulose and not sclerotized; membrane in middle of dorsal surface of endosoma wrinkled and distinctly thicker than other membranous areas (Figure 10K).

#### 3.3.3. Distribution 

China (Nanjing, Taiwan) (Figure 4). 

#### 3.3.4. Measurements

[in mm] ♂(n = 3) Total length to tip of abdomen 23.12–23.64. Length of head (excluding neck) 3.59–3.72; width of head 2.35–2.36; length of anteocular region 1.85–1.87; length of postocular region 0.63–0.68; width of eye 0.79–0.80; synthlipsis 0.74–0,79. Length of antennal segments I–IV = 0.78–0.86/3.77–3.83/-/-; length of visible labial segments I–III = 1.44–1.65/2.09–2.17/0.52–0.58. Length of anterior lobe of pronotum 1.24–1.36; length of posterior pronotal lobe 2.82–2.88; width of anterior pronotal lobe 3.79–3.84; width of posterior pronotal lobe 5.92–6.13. Length of scutellum 2.86–3.03; width of scutellum 2.84–2.98. Length of hemelytron 16.19–16.24. Width of abdomen 9.03–9.45. 

#### 3.3.5. Materials Examined

China: 2♂, Zhiben Hot Spring, Beinan Township, Taitung County, Taiwan, M. Sakai [leg.]; 3~6. V. 1972; T. Esaki [leg.] 8. VIII. 1932; 1♂, Nanjing, Jiangsu, V. 1953. 

### 3.4. Triatoma rubrofasciata (De Geer, 1773) (Figure 11)

#### 3.4.1. Remarks 

**Figure 11:**
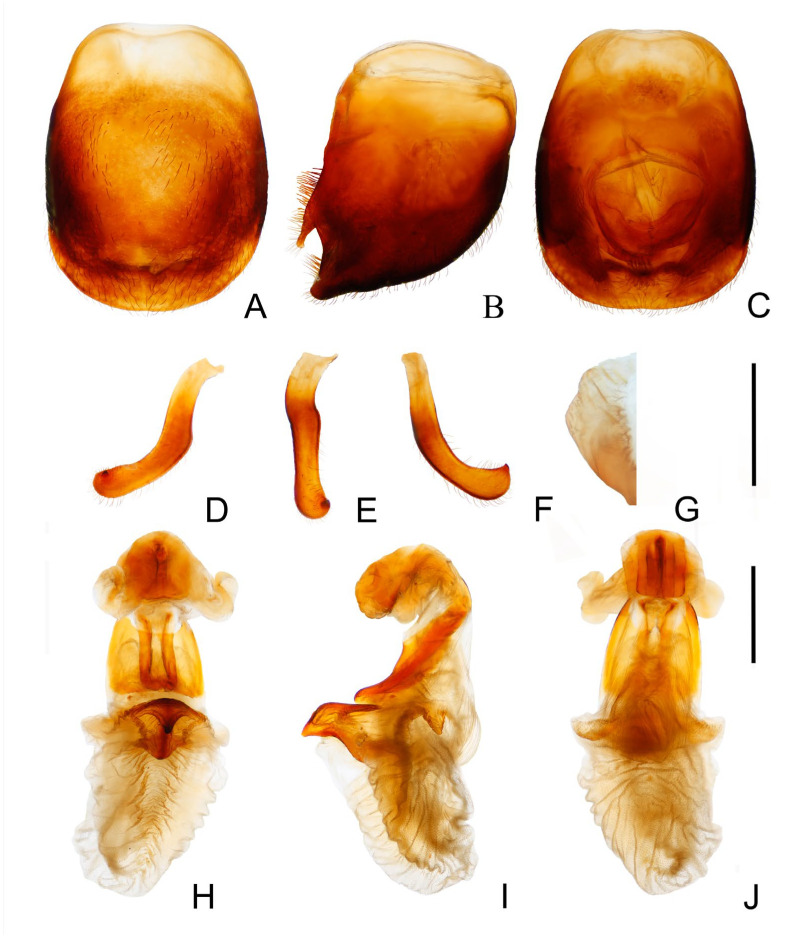
Male external genitalia of *Triatoma rubrofasciata* (De Geer, 1773). (**A**–**C**) pygophore: (**A**) ventral view; (**B**) lateral view; (**C**) dorsal view. (**D**–**F**) paramere: (**D**) dorsal view; (**E**) lateral view; (**F**) ventral view. (**G**) basal lateral sclerite of endosoma. (**H**–**J**) everted phallus: (**H**) dorsal side; (**I**) lateral side; (**J**) ventral side. Scale bars: 1.00 mm (**A**–**F**,**H**–**J**) 0.50 mm (**G**).

The color pattern of *T. rubrofasciata* in China is mostly black, and the lateral margins of the whole pronotum are red and narrow. The forewing has a red strip along vein R+M, and a V-like shape spot on the subapical corium. Images of male genitalia are offered (Figure 11). The medial basal sclerite of phallosoma is strongly sclerotized and shaped like an eagle’s beak (Figure 11H–J). The basal lateral sclerites of the endosoma form an approximate segment of a circle without denticles, and the outline is sinuous (Figure 11G).

#### 3.4.2. Materials Examined

China: 8♂, Zhizhen Village, Baisha Li Autonomous County, Hainan, YS Zhao [leg.], 10. VI. 2017; 5♂, Sanya, Hainan, WH Li, JH Zhang [leg.], 3. V. 1985; 2♂, Yinggeling Natural Reserve, Baisha Li Autonomous County, Hainan, YS Zhao [leg.], 21. VI. 2016; 18. X. 2007; 2♂, Xinglong, Wanning, Hainan, WH Li, JH Zhang [leg.], 24. IV. 1985; 25. IV. 1985; 1♂, Jianfeng Mountain, Ledong Li Autonomous County, Hainan, ZL Wang [leg.], 13. II. 2005; 1♂, Limu Mountain, Qiongzhong Li and Miao Autonomous County, Hainan, 21. IV. 2022; 1♂, Changjiang Li Autonomous County, Hainan; 1♂, Jinjiang, Quanzhou, Fujian, RQ Chen [leg.], 17. VIII. 2006; 1♂, Zhangpu County, Zhangzhou, Fujian, 24. IV. 1963; 1♂, Daming mountain, Nanning, Guangxi, 14. V. 1992; 1♂, Wuming, Nanning, Guangxi, RX Lu [leg.], 11.VI.1980; 1♂, Wuzhou, Guangxi, 20. VIII. 2016; 1♂, Naliang, Fangcheng Gang, Guangxi, 18. IX. 2020; 1♀, Haizhu, Guangzhou, Guangdong, 7. VIII. 2021. 

### 3.5. DNA Barcoding Analysis

A total of 23 mitochondrial COI barcode sequences were generated from 23 species. The DNA barcode region analyzed in this study comprised 658 bp, with no detected insertions, deletions, or presence of stop codons. Nucleotide pair frequency analysis of the entire dataset revealed 371 of 658 (56.4%) sites were conserved; 287 of 658 (43.6%) sites were variable; 229 of 658 (34.8%) sites were parsimony informative; no singleton sites were present. The overall mean nucleotide base frequencies observed for these sequences were T (32.6%), C (21.5%), A (28.7%), and G (17.2%). The average T content was the highest, the average G content was the lowest, and the AT content (61.3%) was higher than the GC content (38.7%) (Table S2). The interspecific genetic distances among 23 species of the genus *Triatoma* were shown in Table S3, and two of these species (*Triatoma picta* Zhao & Cai sp. nov. and *T. atrata* Zhao & Cai sp. nov.) were newly described in this study. The results showed that the distances between *T. picta* Zhao & Cai sp. nov. and other species ranged from 0.14 to 0.28. Similarly, the distances between *T. atrata* Zhao & Cai sp. nov. and others ranged from 0.12 to 0.26. Additionally, the calculated distances between any different *Triatoma* species, except for that between *T. mazzottii* and *T. phyllosoma* (0.003), encompassed a wider range (0.07 to 0.34) than those between *T. picta* Zhao & Cai sp. nov. or *T. atrata* Zhao & Cai sp. nov. and other species. 

### 3.6. Key to the Species of Triatominae from China

1. First antennal segment longer than maxillary plate. Pronotum with reddish lateral margins. Medial basal sclerite of phallosoma eagle’s beak-like shape (Figure 11H–J)......................................................................... *T. rubrofasciata* (De Geer, 1773)
− First antennal segment not longer than maxillary plate. Pronotum with yellowish strips or markings. Medial basal sclerite of phallosoma not eagle’s beak-like shape....................................................................................................... 2
2. Anterolateral angles of pronotum prominent and tips sharp (Figure 2D,E). Bottom third of corium entirely yellowish (Figure 2F,G). Medial basal sclerite of phallosoma subtriangular in the lateral view (Figure 3I). Basal lateral sclerites of endosoma without denticles (Figure 3G)......................................................................... *Triatoma picta* Zhao & Cai sp. nov.
− Anterolateral angles of pronotum slightly prominent and tips round. Not all of bottom third of corium entirely yellowish. Medial basal sclerite of phallosoma oblong in lateral view. Basal lateral sclerites of endosoma with denticles....................................................................................................... 3
3. Width of eye equal to synthlipsis in dorsal view (Figure 9A,E). Body generally brown; rectangular area in middle of each connexival segment black (Figure 8D). Basal lateral sclerites of endosoma sub-semicircular (Figure 10I–J)......................................................................... *Triatoma sinica* Hsiao, 1965
− Width of eye shorter than synthlipsis in dorsal view (Figure 6A). Body generally black; connexivum generally black with joint area of adjacent segments light yellow (Figure 5D). Basal lateral sclerites of endosoma rounded rectangular (Figure 7G) ......................................................................... *Triatoma atrata* Zhao & Cai sp. nov.

## 4. Discussion

The discovery of new distribution localities and previously unknown species has long been of interest to researchers, as it sheds light on their diversity and evolution. Traditionally, species identification and classification have relied on morphological characteristics, such as color pattern, head shape, and genital structure. However, with the help of recent advances in molecular techniques, researchers can potentially obtain more information and tackle intricate problems. In this study, we combined both morphological and molecular approaches to identify and describe or redescribe three Triatominae species. 

The newly described *Triatoma picta* Zhao & Cai sp. nov. is similar to *T. migrans* in color pattern, but there are noticeable differences between these two species [8]. Notably, *T. picta* Zhao & Cai sp. nov. is uniformly dark brown on the dorsal surface of the head, while *T. migrans* features a yellowish stripe in the middle. Additionally, the forewing bottom area of *T. picta* Zhao & Cai sp. nov. is yellowish, whereas *T. migrans*’ is dark brown. The anterolateral angles of *T. migrans* are much longer than those of *T. picta* Zhao & Cai sp. nov. Moreover, *T. migrans* is generally larger than *T. picta* Zhao & Cai sp. nov. in size, although a few exceptions existed. Barcoding analysis reveals that the genetic divergence between *T. picta* Zhao & Cai sp. nov. and *T. migrans* is significant, with a value of 0.17, which exceeds the species delimitation empirical threshold, 0.02 [13]. The genetic distances between *T. picta* Zhao & Cai sp. nov. and other *Triatoma* species are also distinct, with values much higher than 0.02. 

Among the Old-World Triatominae species, *T. atrata* Zhao & Cai sp. nov. stands out due to its unique characteristics. Unlike other species, *T. atrata* Zhao & Cai sp. nov. is generally dark and lacks distinct markings on the connexivum. Regarding morphological structure, *T. sinica* is the species that most resembles *T. atrata* Zhao & Cai sp. nov., while there are notable differences between them. *Triatoma sinica* has much larger eyes, with its width equal to the synthlipsis, and *T. atrata* Zhao & Cai sp. nov. has relatively sharper humeral angles. Moreover, there is a difference in the shape of the basal lateral sclerites of endosoma, with *T. sinica* having sub-semicircle sclerites and *T. atrata* Zhao & Cai sp. nov. having slightly angular ones. The stridulatory sulcus of *T. atrata* Zhao & Cai sp. nov. is wider, and its width-to-length ratio is greater than that of *T. sinica*. The two species also have distinct color patterns. *Triatoma atrata* Zhao & Cai sp. nov. is generally black, and its light yellowish markings are not distinct, while *T. sinica* is brown to dark brown with yellowish stripes and markings. The observed genetic divergences between *T. atrata* Zhao & Cai sp. nov. and the other 22 *Triatoma* species are found to be significantly higher than the empirical threshold, 0.02 [13]. Regrettably, we are unable to calculate the genetic distance between *T. sinica* and *T. atrata* Zhao & Cai sp. nov due to the unavailability of ethanol-preserved specimens and our inability to extract DNA from dry specimens of *T. sinica*. 

Our examination of specimens allowed us to identify three suspected *T. sinica* specimens. Although we did not examine the type specimens, we believe that one of the examined species was collected from the same locality as the type specimens, based on its collection information. Furthermore, we observed that two of the specimens collected in Taiwan were slightly darker than those from Nanjing. After dissecting their genitalia, we discovered that they both have an oblong medial basal sclerite of the phallosoma and lateral sclerites of the endosoma in the form of sub-semicircles with denticles, which are identical to those of specimen from Nanjing. Therefore, we concluded that *T. sinica* was found in a new distribution locality in Taiwan, China.

By combining morphological and molecular methods, we were able to gain a more comprehensive understanding of these species, and this highlights the importance of using multiple approaches to accurately identify and classify Triatominae species.

## Data Availability

The mitochondrial COI barcode sequences of *Triatoma picta* Zhao & Cai sp. nov., *T. atrata* Zhao & Cai sp. nov., *T. migrans*, *T. rubrofasciata* have been deposited in the GenBank under the accession numbers OP062225, OP062226, OP062227, OP062228, respectively.

## Supplementary Materials

The following supporting information can be downloaded at: https://www.mdpi.com/article/10.3390/insects14050450/s1, Table S1: Collecting information of newly sequenced species; Table S2: Nucleotide frequencies of 23 mitochondrial COI barcode sequences; Table S3: Pairwise intra and interspecific genetic distances, based on the Kimura 2-Parameter model.
