# Supplementary material for: Review of Kissing Bugs (Hemiptera: Reduviidae: Triatominae) from China with Descriptions of Two New Species
Source: Insects. 2023 May 10;14(5):450. doi: 10.3390/insects14050450 (PMC10231107; doi:10.3390/insects14050450)
Supplement: Supplementary file 1 [file insects-14-00450-s001.zip › supplementary materials.pdf]

Supplementary Materials

Table S1. Collecting information of newly sequenced species.

Species	Geographical locality	Collection date
Triatoma picta Zhao & Cai, sp. nov.	Jianfeng Mountain, Ledong Li Autonomous County, Hainan, China	26. IV. 2016
Triatoma atrata Zhao & Cai sp. nov.	Binchuan County, Dali Bai Autonomous Prefecture, Yunnan (25.70 °N, 100.55 °E), China	8. VI. 2020
Triatoma migrans Breddin, 1903	Borneo Jungle girl camp, Kinabalu, Malaysia	25. VIII. 2016
Triatoma rubrofasciata (De Geer, 1773)	Zhizhen Village, Baisha Li Autonomous County, Hainan, China	10. VI. 2017

Table S2. Nucleotide Frequencies of mitochondrial COI barcode sequences of *Triatoma*.

Samples	GenBank accession number	T(U)	C	A	G	Total
*T. picta**	OP062225	33.7	19.6	29.3	17.3	658
*T. atrata**	OP062226	35.6	18.7	29.9	15.8	658
*T. migrans**	OP062227	35.0	19.8	28.7	16.6	658
*T. rubrofasciata**	OP062228	33.4	21.1	28.0	17.5	658
T. vandae	KC249392	29.2	25.2	28.6	17.0	658
T. guasayana	MH029699	30.7	24.5	27.2	17.6	658
T. circummaculata	KC249324	30.1	23.4	29.3	17.2	658
T. brasiliensis	KJ580488	29.9	24.3	28.3	17.5	658
T. infestans	NC_035547	30.7	23.4	28.4	17.5	658
T. bolivari	KY033219	32.1	23.1	28.3	16.6	658
T. rubida	MT556664	31.6	21.0	31.0	16.4	658
T. barberi	MT556655	33.0	22.0	27.7	17.3	658
T. protracta	MT556662	35.6	19.0	27.8	17.6	658
T. lecticularia	MT556650	33.9	20.8	28.0	17.3	658
T. gerstaeckeri	MT587783	32.4	20.8	28.6	18.2	658
T. mexicana	NC_050324	31.6	22.0	29.3	17.0	658
T. mazzottii	MT556651	33.1	21.0	28.7	17.2	658
T. phyllosoma	MT556660	33.1	21.0	28.4	17.5	658
T. huehuetenaguensis	MT556649	31.8	22.0	29.3	16.9	658
T. picturata	MT556661	31.9	21.4	29.3	17.3	658
T. sanguisuga	MT556653	31.6	21.9	28.7	17.8	658
T. dispar	MN621059	32.1	22.2	28.6	17.2	658
T. longipennis	KY033220	38.0	17.0	28.3	16.7	658
Avg.		32.6	21.5	28.7	17.2	658
